# Estimation of dietary intake of sodium, potassium, phosphorus and protein in healthy Indian population and patients with chronic kidney disease

**DOI:** 10.3389/fnut.2024.1312581

**Published:** 2024-02-29

**Authors:** Prabhjot Kaur, Ashok Kumar Yadav, Arnab Pal, Ravjit Singh Jassal, Nusrat Shafiq, Nancy Sahni, Vivek Kumar, Vivekanand Jha

**Affiliations:** ^1^Department of Nephrology, Postgraduate Institute of Medical Education and Research, Chandigarh, India; ^2^Department of Experimental Medicine and Biotechnology, Postgraduate Institute of Medical Education and Research, Chandigarh, India; ^3^Department of Biochemistry, Postgraduate Institute of Medical Education and Research, Chandigarh, India; ^4^Department of Pharmacology, Postgraduate Institute of Medical Education and Research, Chandigarh, India; ^5^Department of Dietetics, Postgraduate Institute of Medical Education and Research, Chandigarh, India; ^6^The George Institute for Global Health, UNSW, New Delhi, India; ^7^School of Public Health, Imperial College, London, United Kingdom; ^8^Manipal Academy of Higher Education, Manipal, India

**Keywords:** urinary sodium excretion, salt intake, dietary protein, potassium intake, chronic kidney disease

## Abstract

**Introduction:**

Poor nutritious diet is a major risk element for non-communicable diseases (NCD), which are of considerable public health concern. Given the diverse dietary patterns in India, precise determination of nutrient consumption is crucial for disease management. The present study assessed the dietary intake of sodium, potassium, protein, and phosphorus among North Indians.

**Methods:**

This cross-sectional study included healthy adults and adults with stage 2 to 4 chronic kidney disease (CKD). We analysed sodium, protein, potassium and phosphorus intakes using one-time 24-h urinary excretion. Dietary intake was also analysed in subgroups based on sex, body mass index, blood pressure and abdominal obesity. We evaluated the performance of various equations available to estimate sodium intake using a spot urine sample with respect to the sodium excretion measured in a 24-h urine sample. Descriptive statistics was used along with *t*-test for statistical significance.

**Results:**

A total of 404 subjects (182 adult healthy subjects and 222 adults with CKD) with a mean age of 47.01 ± 11.46 years were studied. Mean dietary intakes of sodium, salt, potassium, protein and phosphorus were 2.94 ± 1.68 g/day, 7.42 ± 4.24 g/day, 1.43 ± 0.59 g/day, 47.67 ± 14.73 g/day and 0.86 ± 0.39 g/day, respectively. There were no differences in nutrient consumption between adults who were healthy and those with CKD. Consumption of sodium, salt, protein, potassium, and phosphorus among healthy population vs. those with CKD were 2.81 ± 1.60 vs. 3.05 ± 1.73 g/day (*p* = 0.152), 7.08 ± 4.04 vs. 7.70 ± 4.37 g/day (*p* = 0.143), 47.16 ± 14.59 vs. 48.08 ± 14.86 g/day (*p* = 0.532), 1.38 ± 0.59 vs. 1.48 ± 0.58 g/day (*p* = 0.087) and 0.86 ± 0.41 vs. 0.87 ± 0.37 g/day (*p* = 0.738), respectively. Men had higher consumption of these nutrients than women. Compared to non-hypertensives, hypertensive subjects had higher consumption of salt (8.23 ± 4.89 vs. 6.84 ± 3.59 g/day, *p* = 0.002) and potassium (1.51 ± 0.63 vs. 1.38 ± 0.55 g/day, *p* = 0.024), however, no difference were found in protein and phosphorus intakes. In terms of performance of equations used to estimate 24-h sodium intake from spot urinary sodium concentration against the measured 24-h urinary sodium excretion, INTERSALT 2 equation exhibited the least bias [1.08 (95% CI, −5.50 to 7.66)].

**Conclusion:**

The study shows higher-than-recommended salt and lower-than-recommended potassium intake in the north Indian population compared to those recommended by guidelines. The dietary protein intake is below the recommended dietary allowance. These findings help the development of targeted policies for dietary modification to reduce the risk of the development and progression of CKD.

## Introduction

The burden of non-communicable diseases (NCDs) such as hypertension, diabetes mellitus, cardiovascular disease (CVD) and chronic kidney disease (CKD) is increasing in India. According to the World Health Organization (WHO), 63% of total deaths reported in 2016 in India were due to NCDs, with 27% attributed to cardiovascular diseases (CVD) (1). Similarly, the number of deaths due to CKD is rising in India. Unhealthy diets, lack of physical activity and use of alcohol and tobacco are major NCD risk factors (2, 3). The WHO guidelines recommend a daily dietary sodium intake of 2 g (corresponding to 5 g salt) and potassium consumption of at least 3.50 g (4). In the presence of chronic conditions such as kidney diseases and hypertension, sodium intake should not be more than 1.50 g per day (5). The National Academies of Sciences, Engineering, and Medicine Dietary Reference Intakes for Sodium and Potassium lists the maximum amount of sodium consumption as 2.30 grams per day for chronic disease risk reduction (6), and the Kidney Disease Global Outcomes (KDIGO) guidelines recommend restricting sodium intake to <2 g/day (7). According to the Institute of Medicine (The National Academy of Medicine), the recommended dietary allowance (RDA) for phosphorus and protein for healthy adults is 700 mg/day and 0.80 g/kg/day (8), respectively. The actual phosphorus and protein intake in the western populations, however, is 1,056–1,617 mg/day (9–11) and 1.30–1.40 g/kg/day (12), respectively. The biological availability of protein depends on the source, with vegetarian sources (biological value 56–74%) being less efficient than animal proteins (biological value 77–104%) (13). Normative data on nutrient intake is limited in Indian populations, and where available, is based on dietary recall, which indicates a gap in knowledge (2, 14–17).

Dietary manipulation is an important strategy in the management of patients with NCDs, including CKD. In addition to salt restriction, protein intake is recommended to not exceed beyond 0.80 g/kg/day (0.55–0.60 g/kg/day for CKD stage 3–5, 0.60–0.80 g/kg/day in case of diabetic patients) to slow the progression of CKD (18). For the Western societies, where the normal dietary protein intake is 1.30 g/kg/day (12), this is commonly expressed as ‘limiting’ protein intake. The appropriateness of this recommendation in other societies with substantially different food habits has not been examined. For example, the usual protein intake in predominantly vegetarian Indian societies is likely lower (39–57 g/day) (2, 14, 16). A systematic review also highlighted the diverse dietary patterns in India, including large variations across regions and over time. For example, sweets, snacks, meat or fish were the most prevalent diets in the Eastern and Southern parts of India whereas fruits, vegetables, rice and pulses were popular in the North and West (19). Accurate assessment of dietary intake is therefore essential to develop appropriate dietary advice for people with chronic conditions. The 24-h urinary excretion method is better than dietary recall methods as recall is a retrospective diet assessment method, where an individual needs to remember their food consumption during the preceding 24 h (20). Also, the recall method is prone to errors due to literacy demand, burden, and challenges with portion size estimation (21).

The dietary protein intake is best estimated by measuring the urea nitrogen appearance rate, which requires a 24-h urine collection (22). Similarly, the estimation of the 24-h urinary sodium excretion is the gold standard for estimating salt intake (23–26), and dietary potassium intake is correlated with the 24-h urinary excretion of potassium (27, 28). Dietary assessment has also been shown to be prone to errors in estimating dietary phosphorus intake, making 24-h urine collection the optimal method for this purpose as well (29).

Information on recommended dietary allowances and actual dietary intake of nutrients are important for development of personalized dietary plans in specific population with variable health issues. There are limited studies with small sample size on healthy individuals that have examined the dietary intake of nutrients. Most of these have focused on salt intake. In this study, we aimed to determine the daily intake of sodium, potassium, protein and phosphorus using 24-h urinary excretion method in a group of healthy subjects and those with mild-to-moderate CKD in north India. Given the importance of ascertaining the sodium intake, we also evaluated the performance of equations designed to estimate dietary sodium intake using sodium concentration in a spot urine sample to help identify the equation best suited for this purpose in Indian subjects. These equations have been derived in Caucasian, Japanese or Chinese populations and use different input parameters, for example, sex and potassium (30–35). None of these were developed for Indians.

## Methods

### Study design and population

**Figure 1:**
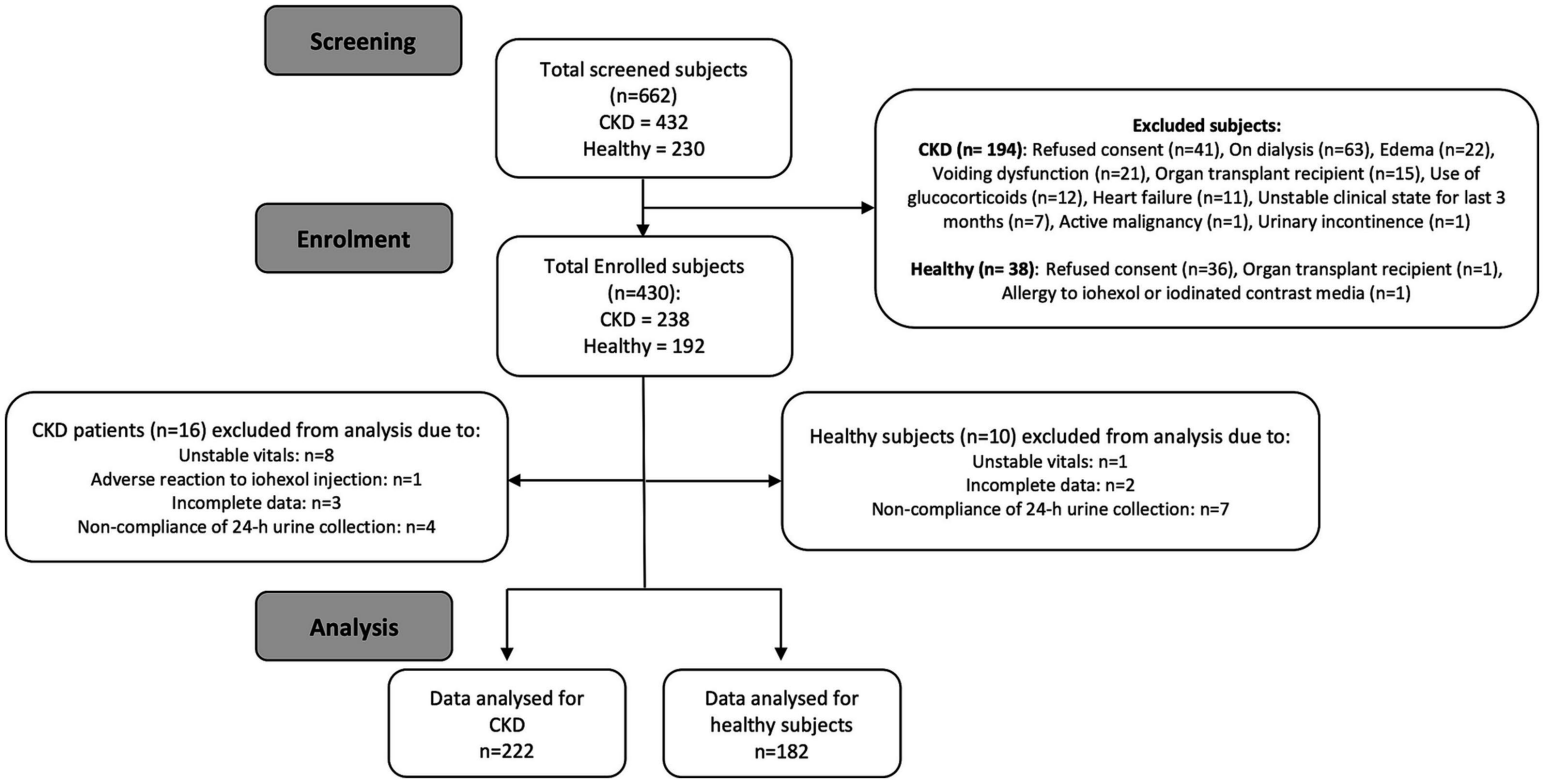
Consort flow diagram for the study subjects.

This cross-sectional study was conducted at the Postgraduate Institute of Medical Education and Research (PGIMER), Chandigarh, India, as a part of the study to measure the glomerular filtration rate in Indian population between 2016 and 2020. We recruited adults over the age of 18 years of either sex with stage 2 to 4 CKD [estimated glomerular filtration rate (eGFR) 15–60 mL/min/1.73m^2^ or eGFR >60 mL/min/1.73m^2^ and proteinuria: ≥500 mg/day] (36) and healthy volunteers drawn from the families of those with CKD. Chronic Kidney Disease-Epidemiology Collaborative Group (CKD-EPI) creatinine equation 2009 (CKD-EPI GFR 2009) was used for eGFR estimation. All healthy volunteers were confirmed to be normotensive, and had a normal HbA1C, eGFR >60 mL/min/1.73 m2, and were normoalbuminuric. Other health conditions were excluded by self-reporting. Individuals with voiding problems, urinary incontinence, limb amputation, chronic liver disease, or cardiac disease were also excluded. Figure 1 shows the flow diagram for the study subjects. The study was approved by the Institutional Ethics Committee at PGIMER, Chandigarh, and all subjects provided written consent. All the methods used in this study were performed in accordance with relevant guidelines and regulations.

### Study conduct

#### Urine sample collection

Written and verbal instructions for 24-h urine collection were provided to all subjects. Subjects were asked to discard the first void of the day of starting the urine collection and collect all subsequent urine voids over the next 24-h period. The volume of 24-h urine samples was measured at reporting. Subjects were asked to collect a second-morning spot urine sample in a 50 mL container. Both spot and 24-h urine samples were transported to the laboratory and aliquots were drawn. Complete urine collection of 24-h urine volume of ≥500 mL was considered for analysis. Urine collection compliance was confirmed by the following formula: collected urine volume/(body weight × 21) > 0.7 (37). For collections with values lower than 0.7, we checked the 24-h urine creatinine excretion. If it was lower than the reference value (< 670 mg for men or < 450 mg for women), the data were excluded from the analysis (37). Urine samples were centrifuged at 2000 rpm for 15 min, and supernatant was collected in 2 mL vials. A total of 4 aliquots of 2 mL were stored at -80°C, out of which one was given to the biochemistry laboratory for analysis. Estimation of dietary intake using 24-h urinary excretion has been considered a reliable tool provided the compliance of 24-h urine collection has been validated which we have done carefully (22).

#### Demographic, anthropometric and clinical measurements

Demographic details were recorded. Weight was measured in minimum clothing using calibrated scales. Height was measured as per standard protocols using a wall mount height measurement scale. The Body Mass index (BMI) was calculated as kg/m^2^ and categorised as BMI ≥ 25 kg/m^2^ and BMI <25 kg/m^2^. The blood pressure was measured using a validated Omron BP monitor following WHO protocol (38). Hypertension was defined as blood pressure ≥ 140/90 mmHg or use of antihypertensive medications. The waist and hip circumferences were measured using standard methods (39). Abdominal obesity was defined as waist circumference ≥ 90 cm in men or ≥ 80 cm in women (40). All these anthropometric measurements, along with blood pressure, was measured three times by a trained study nurse, and the mean of three values was used for analysis.

#### Laboratory analyses

A total 5 mL blood sample was withdrawn from each subject and collected in BD Vacutainer® tubes and serum was separated for the analysis of serum creatinine (modified Jaffe’s method traceable to isotope dilution mass spectrometry (IDMS) standard), urea, and albumin. 24-h urine samples were analysed for sodium, creatinine, protein, urea, phosphorus and potassium. Urine spot samples were analysed for sodium excretion. All investigations were done at the Central Laboratory facility, PGIMER, Chandigarh using COBAS c702 auto-analyzer (Roche Diagnostic Limited, Rotkreuz, Switzerland). The sodium and potassium were measured using Ion-Selective Electrode method, and phosphorus and protein were measured using Molybdate UV (41) and Turbidimetric methods (42) respectively.

Dietary salt intake was calculated using 24-h urine sodium value using the formula (2):

$\begin{array}{l} \mathrm{Amount}\;\mathrm{of}\;\mathrm{salt}\;\left(g/\mathrm{day}\right)\\ =\left(\mathrm{Urinary}\;\mathrm{sodium}\;\left(\mathrm{mmol}/\mathrm{day}\right)\times 0.023\right)/0.397 \end{array}$ .

The dietary protein intake per day was calculated by 24-h urine urea nitrogen method as follows.

$\begin{array}{l} \mathrm{Protein}\;\mathrm{intake}\;\left(g/\mathrm{day}\right)\\ =[\left(\mathrm{urine}\;\mathrm{urea}\;\mathrm{nitrogen}\;\left(g/\mathrm{day}\right)+\left(\mathrm{weight}\;\left(\mathrm{kg}\right)\times 0.031\right)]{\;}^{*}\;6.25\right) \end{array}$ (43, 44).

Potassium intake was calculated as:

$\begin{array}{l} \mathrm{Potassium}\;\mathrm{intake}\;\left(\mathrm{mg}/\mathrm{day}\right)=\mathrm{Urinary}\;\mathrm{potassium}\;\mathrm{excretion}\;\\ \left(\mathrm{mmol}/\mathrm{day}\right)\times 39\;\left(\mathrm{molecular}\;\mathrm{weight}\;\mathrm{of}\;\mathrm{potassium}\right) \end{array}$ (27, 28).

Phosphorus intake was calculated assuming an absorption rate of 65% (29).

$\begin{array}{l} \mathrm{Dietary}\;\mathrm{phosphorus}\;\mathrm{intake}\;\left(\mathrm{mg}/\mathrm{day}\right)\\ =\mathrm{Urinary}\;\mathrm{phosphorus}\;\mathrm{excretion}\;\left(\mathrm{mg}/\mathrm{day}\right)/0.65 \end{array}$ .

##### Estimation of salt intake using spot urinary sodium concentration

We compared the measured 24 h urinary sodium excretion with those derived using seven estimation equations (Kawasaki, Tanaka, Toft, INTERSALT with and without potassium, Whitton and Mage) (30–35). Detailed formulae are presented in Appendix 1 in Supplementary Materials.

### Statistical analyses

Data are presented as mean ± standard deviation or as median and 25th and 75th percentiles. The normality of data was tested using Shapiro–Wilk test. Continuous data were compared using independent t-test or Mann Whitney U test as appropriate. Categorical data were compared using the chi-square test. Correlation between variables was tested using Pearson’s or Spearman’s rho correlation as appropriate. Dietary intake of nutrients was also analysed in subgroups based on BMI, blood pressure and abdominal obesity. Mean bias for 24-h urinary sodium was computed as the difference between the measured and estimated values obtained from different equations and expressed as mean ± standard deviation (SD). The Bland–Altman Plot was used to estimate the bias and limits of agreement between sodium values obtained by 24 h urinary excretion method and sodium estimating equation methods. Limits of agreement were calculated as mean bias ±1.96 × SD. Precision was defined as 95% confidence interval of bias. Two-tailed *p* value <0.05 was considered significant. Data were analyzed using the Statistical Package for the Social Sciences (SPSS) software for Macintosh, version 26.0 (IBM Corp., Armonk, NY, United States).

## Results

Overall, 662 individuals were screened, out of whom 430 were enrolled (Figure 1). Another 26 participants were excluded from analysis because of various reasons and finally, data from 404 participants (182 adult healthy volunteers and 222 adults with CKD) were analyzed (Figure 1). Among adults with CKD, 71 (32%) were in stage 4, 127 (57%) were in stage 3 and 24 (11%) in stage 2. The demographic characteristics of the participants are shown in Table 1. The mean age was 47.01 ± 11.46 years, with equal distribution of men and women. The mean eGFR of the study subjects was 71.10 ± 38.28 mL/min/1,73m^2^, and the mean BMI was 24.94 ± 5.47 kg/m^2^, with 121 (30%) being overweight (BMI ≥25 kg/m^2^) and 51 (12.60%) were obese (BMI ≥30 kg/m^2^).

**Table 1:** Demographic and clinical characteristics of study population.

Parameter(s)	Total (*n* = 404)	CKD (*n* = 222)	Healthy volunteers (*n* = 182)	*p*-value
Age (years)	47.01 ± 11.46	47.82 ± 11.88	46.02 ± 10.88	0.115
Systolic blood pressure (mm Hg)	130 ± 18	135 ± 18	124 ± 17	<0.001
Diastolic blood pressure (mm Hg)	83 ± 11	85 ± 10	80 ± 12	<0.001
Waist/hip ratio	1.08 ± 0.11	1.07 ± 0.11	1.11 ± 0.10	<0.001
BMI (kg/m^2^)	24.94 ± 5.47	25.16 ± 6.23	24.67 ± 4.39	0.364
Hypertension	168 (41.58)	168 (75.66)	—	—
Diabetes mellitus	56 (13.86)	56 (25.230)	—	—
CVD	7 (1.73)	7 (3.15)	—	—
Haemoglobin (g/dL)	12.56 ± 2.01	12.65 ± 2.30	12.45 ± 1.59	0.309
Serum creatinine (mg/dL)	1.46 ± 1.02	2.10 ± 0.95	0.68 ± 032	<0.001
eGFR (ml/min/1.73m^2^)	71.10 ± 38.28	40.77 ± 19.27	107.92 ± 17.78	<0.001
Serum urea (mg/dL)	42.35 ± 27.71	59.04 ± 25.15	21.56 ± 12.50	<0.001
Serum albumin (g/dL)	4.25 ± 0.39	4.18 ± 0.41	4.34 ± 0.33	<0.001
24 h Urine creatinine (mg/day)	869.07 (704.00,1120.84)	934.44 (710.01, 1150.65)	836.92 (691.87, 1028.05)	0.137
24 h Urine protein (mg/day)	143.81 (81.69, 393.68)	303.40 (135.94, 1102.67)	88.50 (62.60, 131.63)	<0.001
Protein intake (g/day)	47.67 ± 14.73	48.09 ± 14.87	47.16 ± 14.59	0.531
Protein intake (g/kg/day)	0.75 ± 0.22	0.73 ± 0.20	0.78 ± 0.25	0.015
24 h urinary sodium (g/day)	2.94 ± 1.68	3.05 ± 1.73	2.81 ± 1.60	0.136
Salt intake (g/day)	7.42 ± 4.24	7.70 ± 4.37	7.08 ± 4.04	0.136
Potassium intake (g/day)	1.43 ± 0.59	1.48 ± 0.58	1.38 ± 0.59	0.095
Phosphorus intake (g/day)	0.86 ± 0.39	0.87 ± 0.37	86 ± 0.41	0.741

BMI: body mass index, eGFR: estimated glomerular filtration rate, CVD; cardiovascular disease expressed as mean ± standard deviation or median (25th and 75th quartiles).

Table 1 shows the daily dietary nutrient intake in the study population. The mean dietary intakes of sodium, salt, potassium, protein and phosphorus were 2.94 ± 1.68 g/day, 7.42 ± 4.24 g/day, 1.43 ± 0.59 g/day, 47.67 ± 14.73 g/day and 0.86 ± 0.39 g/day, respectively. Compared to healthy subjects, those with CKD had similar dietary intakes of sodium (2.81 ± 1.60 g/day vs., 3.05 ± 1.73 g/day, *p* = 0.136), salt (7.08 ± 4.04 g/day vs. 7.70 ± 4.37 g/day, *p* = 0.136) protein (47.16 ± 14.59 g/day vs. 48.08 ± 14.86 g/day, *p* = 0.531), potassium (1.38 ± 0.59 g/day vs1.48 ± 0.58 g/day, *p* = 0.095) and phosphorus (0.86 ± 0.41 mg/day vs. 0.87 ± 0.37 g/day, *p* = 0.741). However, protein intake per kilogram of body weight was more in healthy subjects (0.78 ± 0.25 g/kg/day vs 0.73 ± 0.20 g/kg/day, *p* = 0.015). The salt consumption was above the WHO recommended intake of 5 g/day in 118 (64.83%) of the healthy population and 150 (67.56%) of those with CKD.

In general, men consumed more dietary salt; 8.28 ± 4.44 vs. 6.55 ± 3.83 g/day (*p* < 0.001), protein; 51.43 ± 15.74 g/day vs. 43.84 ± 12.56 (*p* < 0.001), potassium; 1.52 ± 0.58 vs. 1.35 ± 0.58 g/day (*p* = 0.003) and phosphorus; 0.94 ± 0.39 mg/day vs. 0.79 ± 0.36 g/day (*p* < 0.011) as compared to women (Supplementary Table S1).

Stratification based on body mass index (BMI) and blood pressure was done as shown in Table 2. A total of 80 (44%) healthy volunteers and 92 (41%) subjects with CKD were overweight, whereas 168 (76%) of those with CKD were hypertensive. Consumption of salt, potassium, protein and phosphorus was higher among subjects with BMI ≥ 25 than those with BMI < 25, i.e., 8.38 ± 4.67 vs. 6.71 ± 3.73 g/day, *p* < 0.001 (salt), 1.57 ± 0.63 vs. 1.33 ± 0.53 g/day, *p* < 0.001 (potassium), 51.12 ± 13.77 vs. 45.11 ± 14.92 g/day, *p* < 0.001 (protein) and 0.93 ± 0.38 vs. 0.82 ± 0.38 g/day, *p* = 0.005 (phosphorus), respectively. Compared to normotensive study subjects, hypertensive individuals (blood pressure ≥ 140/90 mm Hg) consumed more salt and potassium, i.e., 8.23 ± 4.89 vs. 6.84 ± 3.59 g/day (*p* = 0.002) and 1.51 ± 0.63 vs. 1.38 ± 0.55 g/day (*p* = 0.024), respectively. No differences were seen in terms of protein and phosphorus intake.

**Table 2:** Daily dietary intake of nutrients per 24 h of urinary excretion as per BMI and blood pressure category in different subgroups.

	BMI (kg/m^2^)			Blood pressure (mm Hg)		
	≥25	<25	*p* value	≥140/90	<140/90	*p* value
**Total**						
Number of subjects	172	232		168	236	
Salt (g/day)	8.38 ± 4.67	6.71 ± 3.73	<0.001	8.23 ± 4.89	6.84 ± 3.59	0.002
Protein (g/day)	51.12 ± 13.77	45.11 ± 14.92	<0.001	48.69 ± 14.32	46.94 ± 15.00	0.236
Protein (g/kg/day)	0.70 ± 0.16	0.79 ± 0.25	<0.001	0.75 ± 0.20	0.76 ± 0.24	0.594
Potassium (g/day)	1.57 ± 0.63	1.33 ± 0.53	<0.001	1.51 ± 0.63	1.38 ± 0.55	0.024
Phosphorus (g/day)	0.93 ± 0.38	0.82 ± 0.38	0.005	0.89 ± 0.39	0.85 ± 0.38	0.337
**Men**						
Number of subjects	76	128		92	112	
Salt (g/day)	9.80 ± 4.80	7.37 ± 3.97	<0.001	9.51 ± 5.16	7.27 ± 3.46	<0.001
Protein (g/day)	56.53 ± 15.06	48.39 ± 15.39	<0.001	53.07 ± 14.63	50.07 ± 16.53	0.172
Protein (g/kg/day)	0.71 ± 0.16	0.79 ± 0.26	0.004	0.75 ± 0.20	0.77 ± 0.26	0.702
Potassium (g/day)	1.75 ± 0.63	1.39 ± 0.52	<0.001	1.66 ± 0.65	1.41 ± 0.50	0.003
Phosphorus (g/day)	1.05 ± 0.40	0.87 ± 0.38	0.003	0.96 ± 0.39	0.91 ± 0.39	0.372
**Women**						
Number of subjects	96	104	0.013	76	124	0.701
Salt (g/day)	7.25 ± 4.27	5.90 ± 3.26		6.68 ± 4.08	6.46 ± 3.68	
Protein (g/day)	46.83 ± 10.96	41.07 ± 13.32	0.001	43.39 ± 12.05	44.11 ± 12.89	0.691
Protein (g/kg/day)	0.69 ± 0.16	0.79 ± 0.24	0.001	0.73 ± 0.20	0.75 ± 0.22	0.646
Potassium (g/day)	1.44 ± 0.60	1.27 ± 0.55	0.037	1.34 ± 0.56	1.35 ± 0.58	0.914
Phosphorus (g/day)	0.83 ± 0.34	0.75 ± 0.38	0.129	0.79 ± 0.37	0.79 ± 0.36	0.951

BMI; body mass index.

Also, data were grouped based on the presence or absence of sex-specific abdominal obesity (waist circumference ≥ 90 cm in men or ≥ cm in women) (40). A total of 112 (62%) healthy individuals, and 137 (62%) of those with CKD had abdominal obesity. Overall, 115 (57%) men and 134 (68%) women had abdominal obesity. The protein intake in men and women with abdominal obesity was higher than in the non-obese (abdominal) individuals (54.33 ± 15.12 vs. 47.62 ± 15.98 g/day, *p* = 0.003 for men; and 45.68 ± 11.50 vs. 39.60 ± 14.05 g/day, *p* = 0.004 for women). However, weight-corrected protein intake was lower in men and women with abdominal obesity (0.72 ± 0.18 vs. 0.81 ± 0.28 g/kg/day, *p* = 0.014 for men and 0.71 ± 0.18 vs. 0.80 ± 0.27 g/kg/day, *p* = 0.019 for women). Intake of salt, potassium and phosphorus was significantly higher in both men and women with abdominal obesity (Table 3).

**Table 3:** Daily dietary intake of nutrients as per abdominal obesity in men and women.

Abdominal obesity			
**Men**			
**Waist circumference (cm)**	**≥90**	**<90**	***p* value**
Number of subjects	115	87	
Salt (g/day)	8.86 ± 4.88	7.57 ± 3.69	0.033
Protein (g/day)	54.33 ± 15.12	47.62 ± 15.98	0.003
Protein (g/kg/day)	0.72 ± 0.18	0.81 ± 0.28	0.014
Potassium (g/day)	1.62 ± 0.63	1.39 ± 0.49	0.005
Phosphorus (g/day)	0.98 ± 0.38	0.88 ± 0.40	0.091
**Women**			
**Waist circumference (cm)**	**≥80**	**<80**	***p* value**
Number of subjects	134	62	
Salt (g/day)	6.95 ± 3.95	5.63 ± 3.42	0.019
Protein (g/day)	45.68 ± 11.50	39.60 ± 14.05	0.004
Protein (g/kg/day)	0.71 ± 0.18	0.80 ± 0.27	0.019
Potassium (g/day)	1.41 ± 0.56	1.21 ± 0.59	0.025
Phosphorus (mg/day)	0.83 ± 0.33	0.70 ± 0.43	0.040

**Figure 2:**
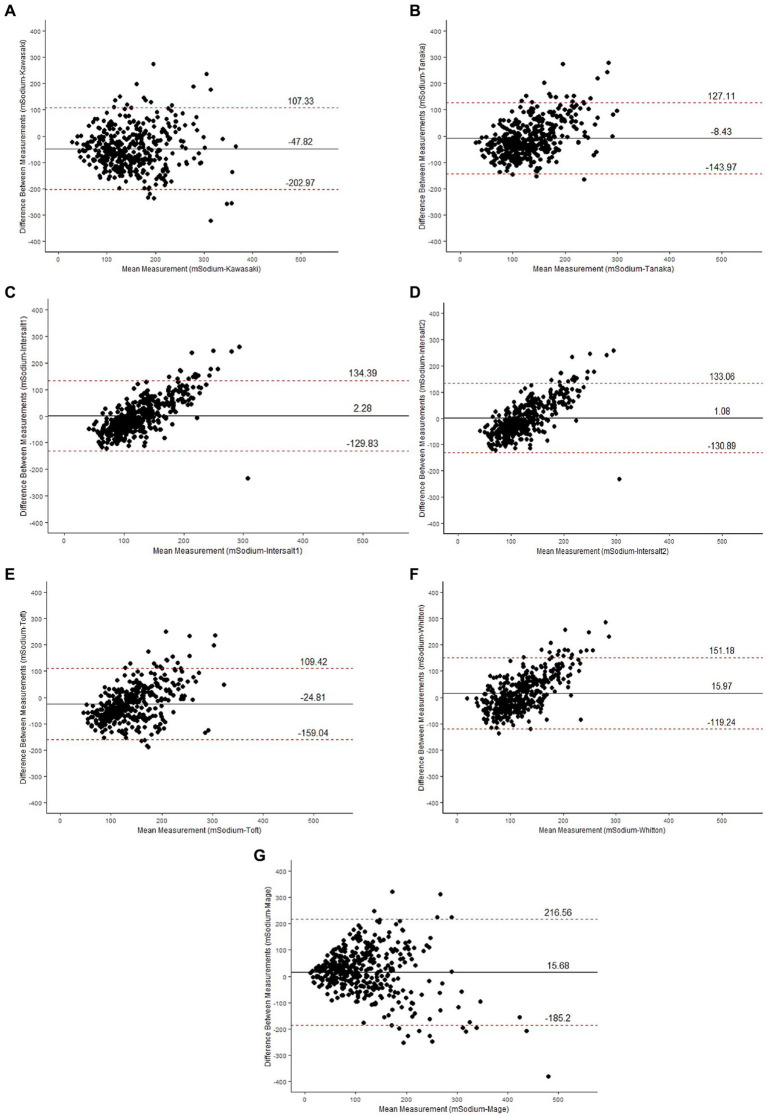
Bland–Altman plots for the difference in agreement between the measured sodium (mSodium) and estimated sodium using Kawasaki **(A)**, Tanaka **(B)**, INTERSALT1 **(C)**, INTERSALT1 **(D)**, Toft **(E)**, Whitton **(F)** and Mage **(G)** estimating equations shown by plotting the bias (measured sodium intake minus estimated sodium intake) against the mean of measured and estimated sodium intake. Central line represent the mean bias between measured and estimated methods and dotted line represent ±1.96 SD.

Table 4 and Figure 2 show the performance of equations used to estimate 24-h sodium intake from spot urinary sodium concentration against the measured 24-h urinary sodium excretion. The mean bias ranged from −47.82 (95% CI, −55.54 to −40.09) for the Kawasaki equation to 15.97 (95% CI, 9.24 to 22.69) for the Whitton formula. The INTERSALT2 formula had the least bias of all equations [1.08 (95% CI, −5.50 to 7.65)].

**Table 4:** Comparison of measured and estimated 24 h urinary sodium excretion in study subjects.

Method	Bias (Mean ± SD)	95% CI	LOA
Kawasaki	−47.82 ± 79.16	−55.54 to −40.09	107.33 to −202.97
Tanaka	−8.43 ± 69.16	−15.17 to −1.69	127.11 to −143.97
INTERSALT1	2.28 ± 67.40	−4.29 to 8.85	134.39 to −129.83
lNTERSALT2	1.08 ± 67.33	−5.50 to 7.65	133.06 to −130.89
Toft	−24.81 ± 68.48	−31.49 to −18.13	109.42 to −159.04
Whitton	15.97 ± 68.99	9.24 to 22.69	−119.24 to 151.18
Mage	15.68 ± 102.49	5.69 to 25.68	216.56 to −185.2

SD: standard deviation, CI: confidence interval, LOA: limit of agreement.

## Discussion

This is the first study to provide a comprehensive assessment of the intake of multiple nutrients in the north Indian population that included healthy subjects and those with CKD. We document a higher-than-recommended dietary intake of salt and phosphorus and relatively low protein and potassium consumption.

**Figure 3:**
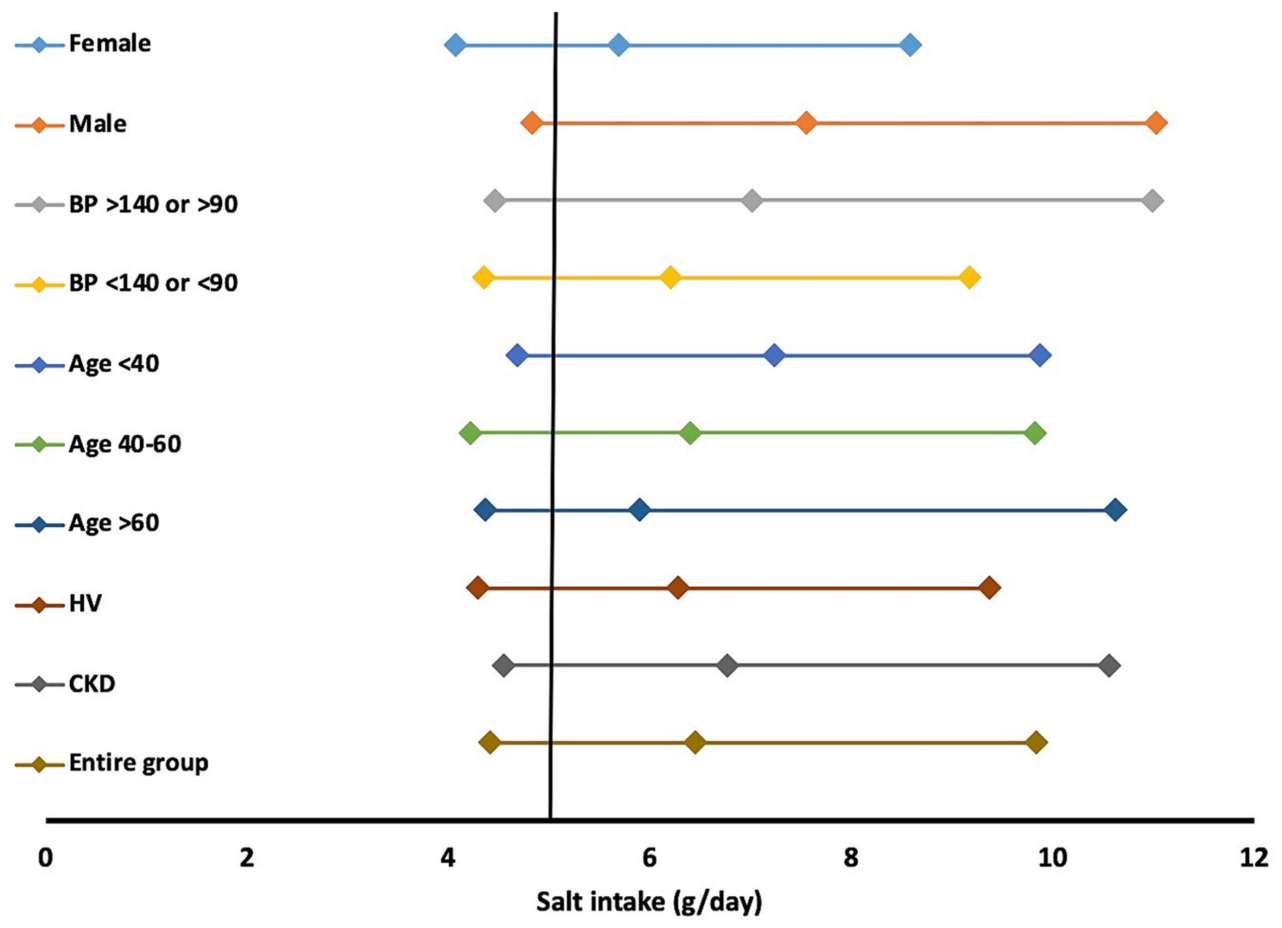
Salt intake per day in various subgroups. Data represented as median (25th, 75th percentile). HV; healthy volunteer, CKD; chronic kidney disease.

The dietary salt intake was higher than recommended in about two-thirds of the study subjects, with no heterogeneity with regard to sex, or presence of hypertension or CKD (Figure 3). While this is the first study looking at salt intake in subjects with CKD, a similar finding has been reported in general population studies conducted in different parts of India. Johnson et al., using 24-h estimation, found the dietary salt intake to be higher among the population residing in Andhra Pradesh (9.46 g/day) compared to people of Delhi and Haryana (8.59 g/day) (17). A nationally representative cross-sectional survey conducted in 2017–18 in India estimated the salt intake to be 8 g/day using the INTERSALT equation on spot urine samples (45). The somewhat lower salt intake amongst subjects in this study compared to those from older studies could be attributed to increased awareness about the benefit of salt reduction and human health, in particular the targeted advice received by patients with CKD, which might have also influenced the consumption in other members of the family from whom the healthy population was drawn. Although the direction of movement is encouraging, a majority of the population (including those with CKD) is still consuming higher than recommended amounts of salt, indicating an unfinished public health agenda. This might also indicate the limits to which salt reduction is possible only with education. A study from China also reported the consumption of similar amounts of salt (7.40 g/day) among patients with CKD (46). Together, these findings raise the need to consider alternate interventions, such as low-sodium salt substitutes. Concerns are often raised about the high potassium content of salt substitutes. While these may be valid for those with advanced stages of CKD, large population-based studies (47) have not shown an increased risk of hyperkalemia. Given the remarkable ability of the kidneys to excrete potassium until the late stages of CKD, it is unlikely that use of salt substitutes will pose a substantial risk of hyperkalemia at a population level (48). Appropriate caution, however, should be exercised in those with advances stages of CKD.

Also striking was the low potassium intake in the study population, not only amongst those with CKD but also in the otherwise healthy population. The intake was substantially lower than 3.50 g/day recommended by the WHO for the healthy population and 2-4 g for those with early stages of CKD as per KDOQI 2020 guidelines (49). Given that nuts, green vegetables, and fruits are the main sources of potassium in the diet, the finding reflects low dietary diversity in our population (50). Our findings are consistent with the results of a 32-country INTERSTROKE study that found overall low urinary potassium excretion (1.68 g/day) in 32 countries, including India (15), and other Indian studies (50, 51). Studies from West Africa (1.8 g/day) (52) and China (1.5 g/day) (53) reported potassium intake similar to the current study. The combination of higher than recommended sodium and lower than recommended potassium collectively intake raises cardiovascular disease risk.

Another notable finding was the relatively low dietary protein intake, especially in the healthy individuals (0.78 g/kg). Previous studies from different parts of India that included healthy people and those with hypertension, CKD and diabetes have shown similar findings (Table 5). Protein intake measured using 24-h or 3-day dietary recall and or a validated Food Frequency Questionnaire (FFQ) in healthy individuals varies from 39.54 g/day to 67.5 g/day (2, 14, 16, 58), whereas protein intake in subjects with CKD was 39.05 g/day (2, 14, 16, 58). This finding is of relevance with regard to the CKD population. Current guidelines recommend restricting dietary protein intake to about 0.8 g/kg/day in those with CKD stage 1–2 and 0.55–0.60 g/kg/day in those with CKD Stage 3–5 (17) to retard disease progression. Data from Western countries show a wide variation in protein intake. A Danish survey reported protein intake ranging from 75.5–94.2 g/day among men and 59.1–69.7 g/day among women (59). A multinational (Denmark, Finland, Netherlands, United Kingdom, Spain, Bulgaria, Australia, New Zealand) randomised controlled trial in prediabetic obese individuals showed a protein intake of 105.8 ± 35.7 g/day (60, 61). The Nurses’ Health Study from the US reported a mean protein intake of 76 grams both in subjects with normal renal function and with mild renal insufficiency (62). The EPIC-Oxford cohort of the general population, which included meat eaters, fish eaters, vegetarians, and vegans, also showed high protein intakes (0.99 to 1.28 g/kg/day) (63). Therefore in the Western context, restriction of dietary protein intake is advised. However, this advice is interpreted in other parts of the world without contextualizing the prevalent dietary practice. Further, excessive protein restriction is likely to lead to protein-energy wasting, a phenomenon shown to be associated with poorer outcomes in those with CKD (64).

**Table 5:** Studies showing the dietary intake of protein, salt, potassium and phosphorus in Indians.

Studies	Place	Number of subjects	Age group (years)	Men/Women	Population	Method used	Protein (g/day)	Salt (g/day)	Potassium (g/day)	Phosphorus (g/day)
Mathur et al. (45)	India	2,266	18–69	1081/1185	General community population	INTERSALT equation with Potassium		8.00		
Thakur et al. (54)	Haryana	5,078	18–69	2294/2784	General community population	Diet and dietary salt questionnaire		8.00		
Johnson et al. (17)	Delhi, Haryana, and Andhra Pradesh	1,395	>20	704/691	General community population	24 h urinary sodium excretion		9.46 (Andhra Pradesh) 8.59 (Delhi and Haryana)		
Kumbla et al. (55)	Delhi, Mumbai, Calcutta, Bangalore and Chennai	466	18–75	254/192	Patients with HTN and dyslipidaemia	Three-day dietary recall		14.13 (Delhi) 9.81 (Mumbai) 10.12 (Calcutta) 9.38 (Bangalore and Chennai)		
Smina et al. (2)	Chennai	200	25–70	111/89	Healthy controls, patients with T2DM, HTN and CKD	24-h dietary recall method for protein intake, 24 h urinary sodium excretion for salt intake	39.54 (HC) 39.50 (T2DM) 42.9 (HTN) 39.05 (CKD)	12.60 (HC) 14.52 (T2DM) 11.85 (HTN) 13.37 (CKD)		
Radhika et al. (56)	Chennai	1902	>20	821/1081	Healthy controls and patients with HTN	Semi quantitative food frequency questionnaire		8.00 (HC) 9.90 (HTN)		
Anand et al. (50)	Delhi, Haryana	1,397	>20	624/773	General community population	24 h potassium excretion			1.14 (Rural) 1.08 (urban)	
Berkemeyer et al. (57)	Delhi	10	20–50		Healthy controls	2-day dietary record	67.90			1.79
Joshi et al. (16)	India	794	≥18	370/424	Gen population	3-day dietary recall, and a validated Food Frequency Questionnaire (FFQ)	57.89			
Kumar et al. (14)	Delhi	255	20–69	0/255	Healthy women	24-h dietary recall method	48.70			

CKD: chronic kidney disease, HC: healthy controls, HTN: Hypertension, T2DM: Type 2 diabetes.

Regarding phosphorus intake, the mean intake was above the recommended levels. Usually, the dietary phosphorus intake tracks with the protein in the western meat-based diets, but there may be a divergence in India, where dairy products are the main source of phosphorus and a reduced phosphorus intake in those with CKD could indicate preferential reduction of dairy products. Finally, doubts have been raised about the reliability of 24-h urinary phosphate for estimating dietary intake in the presence of CKD (65).

We have summarised findings from published studies on dietary intakes of multiple nutrients across India including healthy and individuals with various NCDs (Table 5) (2, 14, 16, 17, 45, 50, 54–57).

The salt, protein, potassium and phosphorus intakes were higher among subjects with higher BMI than those with normal BMI, consistent results with earlier findings (66–70), suggesting overall better nourishment. In line with results as per BMI, both men and women subjects with abdominal obesity has higher intake of all these nutrients. We also noted some sex-specific differences in dietary intake of salt, protein, potassium, and phosphorus. All of them were significantly lower in females. These findings are consistent with previous studies, and indicate a comparative nutritional disadvantage for females, except for salt.

Finally, we analyzed the performance of the estimated 24-h sodium consumption by comparing it with 24-h urine sodium excretion levels. Our data shows that the INTERSALT2 exhibited the least bias. Hence, we suggest the use of this equation to estimate sodium intake using spot urine samples. Whitton et al. also found the INTERSALT equation suitable for estimating sodium excretion in urban Asian populations (Singapore residents of Chinese, Malay, and Indian ethnicity) (30). One possible reason this equation performed better in our population is that it was developed using data from 32 countries that included Indian subjects in contrast to others that did not include Indian subjects. A recent study, however, found poor agreement between the actual sodium intake and the estimated intake using all equations, suggesting a high degree of variability and the need to use data from estimation equations with caution (71).

The strength of this study is the inclusion of a well-phenotyped population, including healthy subjects and those with CKD and the use of 24-h urine excretions for analysis of nutrient intake. Our study had a few limitations, including a relatively small sample size, lack of adjustments for dietary energy intake, lack of dietary assessment over a period of time and lack of a community-based study sample as the study was conducted at the hospital level. We also did not correct for the faecal loss of potassium and phosphorus. This limits the value of using urinary excretion as a surrogate for intake. Further, The enrolment of healthy subjects from amongst the family members of subjects with CKD might have introduced a bias since they could have altered their dietary habits as well. Therefore, our findings remain hypothesis-generating for the general population and should be confirmed in larger population-based samples.

In conclusion, the study shows that intake of several nutrients, including salt, potassium, phosphorus and protein, is either above or below the recommended levels, which might be one reason for increased NCD risk, including CKD. An improved understanding of dietary patterns and nutrient intakes will help provide tailored dietary advice to persons with various health conditions, including CKD. These data can help support the development of locally appropriate guidelines and implementation strategies such as public awareness, counselling at the individual level, and developing appropriate food policy.

## Data availability statement

The original contributions presented in the study are included in the article/Supplementary material, further inquiries can be directed to the corresponding authors.

## Ethics statement

The studies involving humans were approved by Postgraduate Institute of Medical Education and Research, Chandigarh, India. The studies were conducted in accordance with the local legislation and institutional requirements. The participants provided their written informed consent to participate in this study.

## Author contributions

PK: Data curation, Methodology, Writing – original draft. AY: Conceptualization, Data curation, Formal analysis, Funding acquisition, Methodology, Project administration, Supervision, Validation, Writing – original draft, Writing – review & editing. AP: Investigation, Methodology, Writing – review & editing. RJ: Investigation, Writing – review & editing. NuS: Methodology, Supervision, Resources, Writing – review & editing. NaS: Methodology, Data curation, Writing – review & editing. VK: Funding acquisition, Investigation, Data curation, Resources, Supervision, Writing – review & editing. VJ: Conceptualization, Data curation, Funding acquisition, Resources, Supervision, Writing – review & editing.
