# Supplementary material for: Estimation of dietary intake of sodium, potassium, phosphorus and protein in healthy Indian population and patients with chronic kidney disease
Source: Front Nutr. 2024 Feb 29;11:1312581. doi: 10.3389/fnut.2024.1312581 (PMC10937368; doi:10.3389/fnut.2024.1312581)
Supplement: Supplementary file 1 [file Data_Sheet_1.PDF]

Supplementary Material

Estimation of dietary intake of sodium, potassium, phosphorus and protein in healthy Indian population and patients with chronic kidney disease

Prabhjot Kaur[1], Ashok Kumar Yadav[2], Arnab Pal[3], Ravjit Singh Jassal[3], Nusrat Shafiq[4], Nancy Sahni[5], Vivek Kumar[1], Vivekanand Jha[6,7,8]

[1]Department of Nephrology, Postgraduate Institute of Medical Education and Research, Chandigarh, [2]Department of Experimental Medicine and Biology , Postgraduate Institute of Medical Education and Research, Chandigarh, [3]Department of Biochemistry , Postgraduate Institute of Medical Education and Research, Chandigarh, [4]Department of Pharmacology , Postgraduate Institute of Medical Education and Research, Chandigarh, [5]Department of dietetics, Postgraduate Institute of Medical Education and Research, Chandigarh, [6]The George Institute for Global Health, New Delhi , [7]School of Public Health, Imperial College, London, UK, [8]Manipal Academy of Higher Education, Manipal, India

Appendix – I

Equations used for estimation of salt intake using spot urinary sodium excretion

Equation	
Kawasaki	Male: $16.3\times[Na_{SU}/Cr_{SU}\times1/10\times(7.39\times height+15.12\times weight-12.63\times age-79.9)]^{0.5}$<br>Female: $16.3\times[Na_{SU}/Cr_{SU}\times1/10\times(5.09\times height+8.58\times weight-4.72\times age-74.95)]^{0.5}$
Tanaka	$21.98\times[Na_{SU}/Cr_{SU}\times1/10\times(16.14\times height+14.89\times weight-2.04\times age-2244.45)]^{0.392}$
Toft	Male: $33.56\times[Na_{SU}/Cr_{SU}\times1/10\times(-7.54\times Age+14.15\times weight+3.48\times height+423.15)]^{0.345}$<br>Female: $52.65\times[Na_{SU}/Cr_{SU}\times1/10\times(-6.13\times Age+9.97\times weight+2.45\times height+342.73)]^{0.196}$
INTERSALT1	Male: $(0.46\times Na_{SU}+25.46)-2.75\times Cr_{SU}-0.13\times K_{SU}+4.10\times BMI+0.26\times Age$<br>Female: $(0.34\times Na_{SU}+5.07)-2.16\times Cr_{SU}-0.09\times K_{SU}+2.39\times BMI+2.35\times Age\ 0.03\times Age^{2}$
INTERSALT2	Male: $(0.45\times Na_{SU}+23.51)-3.09\times Cr_{SU}+4.16\times BMI+0.22\times Age$<br>Female: $(0.33\times Na_{SU}+3.74)-2.44\times Cr_{SU}+2.42\times BMI+2.34\times Age-0.03\times Age^{2}$
Whitton	Male: $88.66+0.55\times Na_{SU}-1.34\times Cr_{SU}-1.05\times K_{SU}-0.87\times Age+2.10\times BMI+39.30$<br>Female: $88.66+0.55\times Na_{SU}-1.34\times Cr_{SU}-1.05\times K_{SU}-0.87\times Age+2.10\times BMI$
Mage	Male: $Na_{SU}/(Cr_{SU}\times10)\times[0.00179\times(140-Age)\times weight^{1.5}\times height^{0.5}$<br>Female: $Na_{SU}/(Cr_{SU}\times10)\times[0.00163\times(140-Age)\times weight^{1.5}\times height^{0.5}$

Na_{SU}, Spot urinary sodium; K_{SU}, Spot urinary potassium; Cr_{SU}, Spot urinary creatinine

Table 1. Demographic characteristics and clinical parameters in study population

Parameter(s)	Men (n=204)	Women (n=200)	P value
Age (years)	49.27±12.01	44.70±10.40	<0.001
Systolic blood pressure (mmHg)	133±18	127±18	<0.001
Diastolic blood pressure (mmHg)	84±11	82±12	0.027
Waist/hip ratio	1.04±0.09	1.12±0.10	<0.001
BMI (kg/m^2)	24.67±6.28	25.21±4.48	0.306
Haemoglobin (g/dL)	13.15±2.12	11.96±1.69	<0.001
Serum creatinine (mg/dL)	1.84±1.01	1.07±0.88	<0.001
eGFR (ml/min/1.73m^2)	56.59±	85.82±38.33	<0.001
Serum urea (mg/dL)	51.70±29.31	32.56±22.10	<0.001
Serum albumin (g/dL)	4.25±0.43	4.26±0.34	0.919
24h Urine creatinine (mg/day)	1010.61 (825.05, 1244.53)	757.66 (604.26, 904.79)	<0.001
24h Urine protein (mg/day)	179.77 (105.03, 639.81)	110.73 (70.36, 234.93)	<0.001
Protein intake (g/day)	51.43±15.74	43.84±12.56	<0.001
Protein intake (g/kg/day)	0.76±0.23	0.74±0.21	0.389
24h urinary Sodium (g/day)	3.28±1.76	2.60±1.52	<0.001
Salt intake (g/day)	8.28±4.44	6.55±3.83	<0.001
Potassium intake (g/day)	1.52±0.58	1.35±0.58	0.003
Phosphorus intake (g/day)	0.94±0.39	0.79±0.36	<0.001

BMI: body mass index, eGFR: estimated glomerular filtration rate
Expressed as mean ± standard deviation or median (25th and 75th quartiles)
